# Supplementary material for: Mitochondrial metabolism sustains DNMT3A-R882-mutant clonal haematopoiesis
Source: Nature. 2025 Apr 16;642(8067):431–41. doi: 10.1038/s41586-025-08980-6 (PMC12158785; doi:10.1038/s41586-025-08980-6)
Supplement: Supplementary file 1 — Supplementary Figs. 1–8. [file 41586_2025_8980_MOESM1_ESM.pdf]

nature portfolio

https://doi.org/10.1038/s41586-025-08980-6

Supplementary information

Mitochondrial metabolism sustains *DNMT3A*-R882-mutant clonal haematopoiesis

In the format provided by the authors and unedited

Supplementary Figure 1

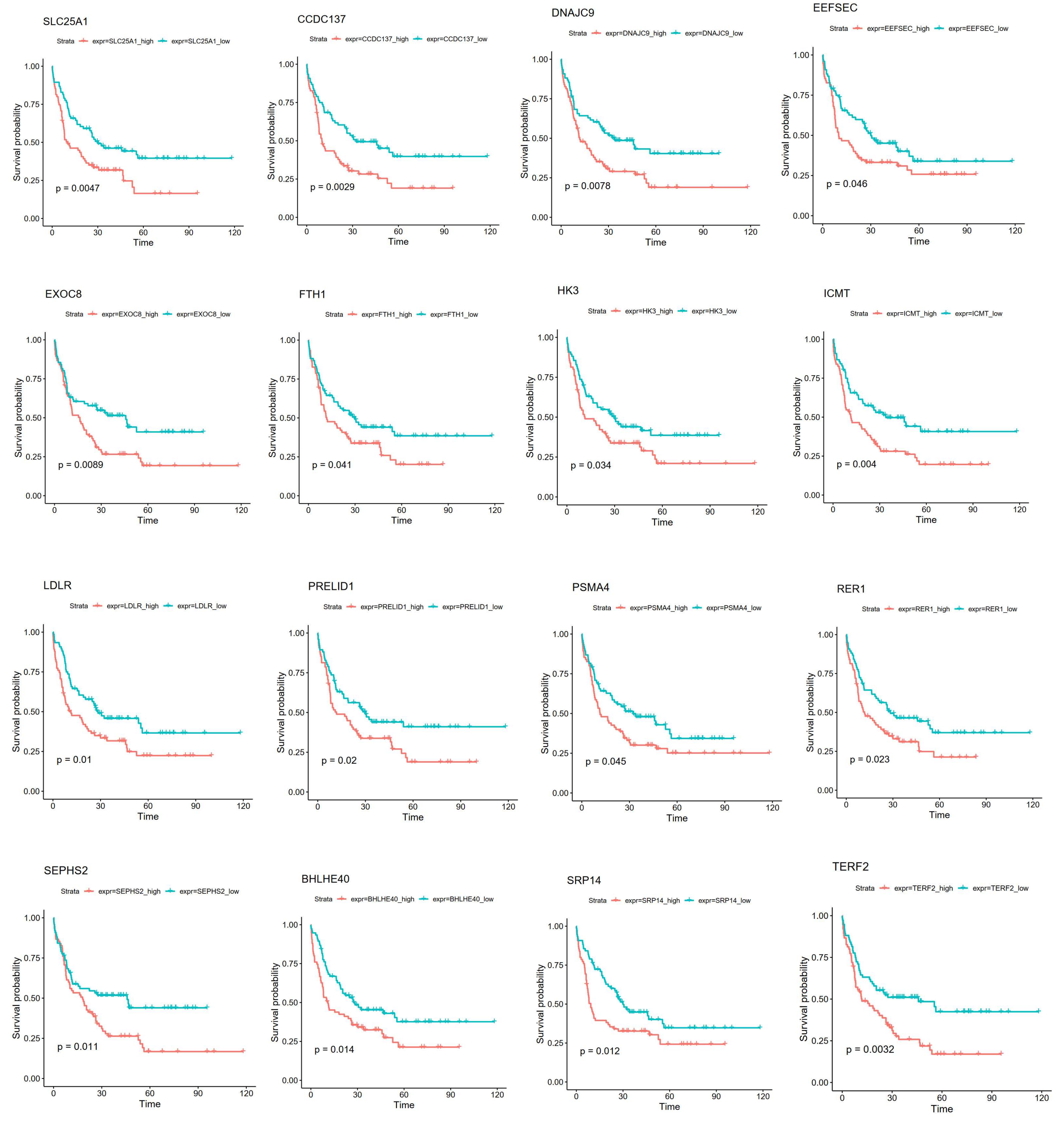

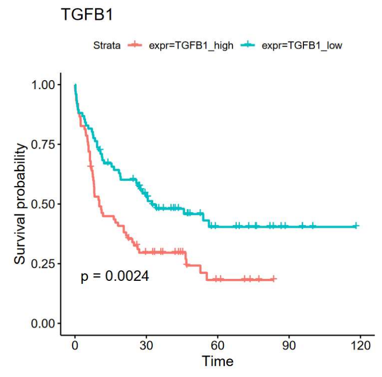

Supplementary Figure 1. *Dnmt3a*$^{R882H/+}$ - specific vulnerability genes whose expression correlates with worse AML patient survival.

TCGA AML data were used to determine the corelation between expression of selected genes and disease-free survival of AML patients. Patients were dichotomised according to gene expression levels, as below and above median, with the survival of the two groups compared using Kaplan-Meier analysis, with log-rank test.

Supplementary Figure 2

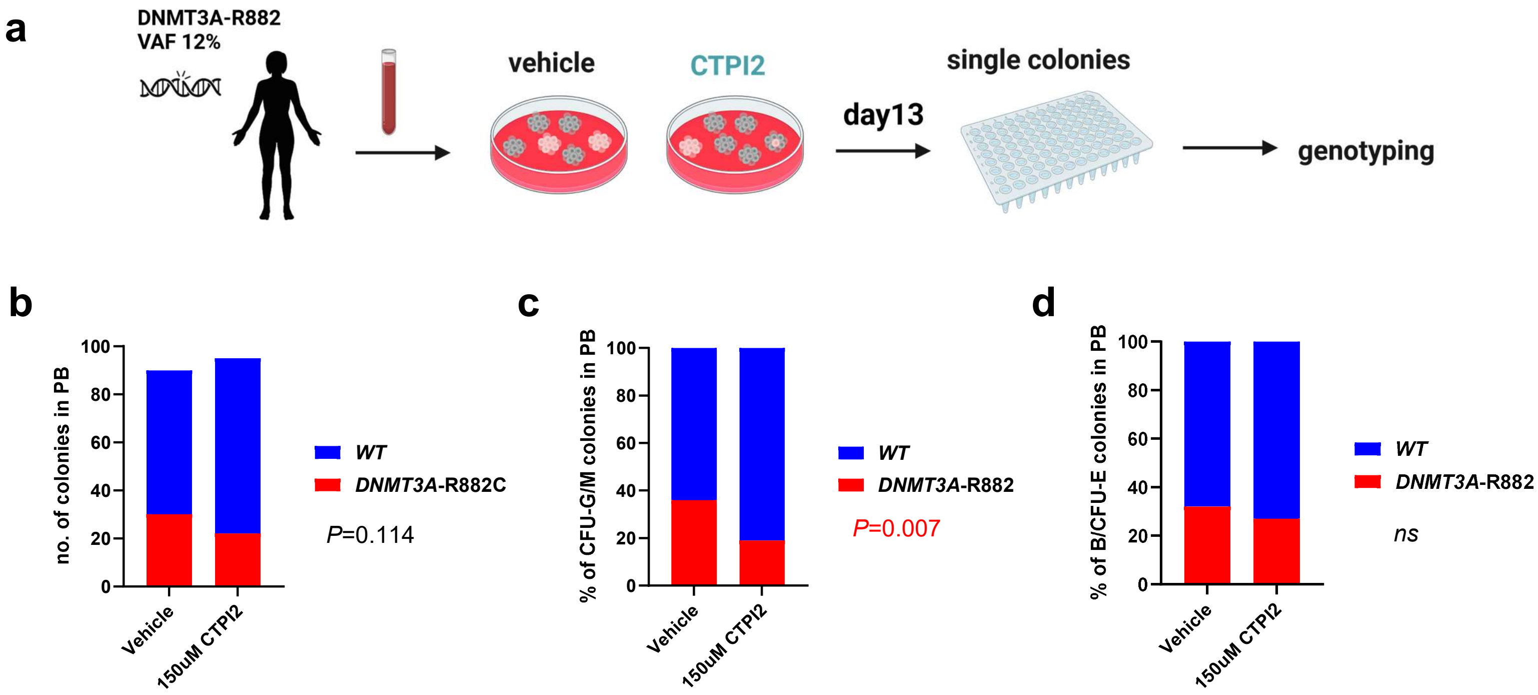

Supplementary Figure 2. Impact of CTPI2 on human DNMT3A-R882 mutant HSPC colony formation

(**a**) Schematic representation of the CTPI2 validation strategy in human primary DNMT3A-R882 CH (**b**) Quantification of the proportion of all (**c**) CFU-G/M and (**d**) B/CFU-E colonies in CTPI2 and vehicle treated cells. P by two-sided Chi-square test.

Supplementary Figure 3

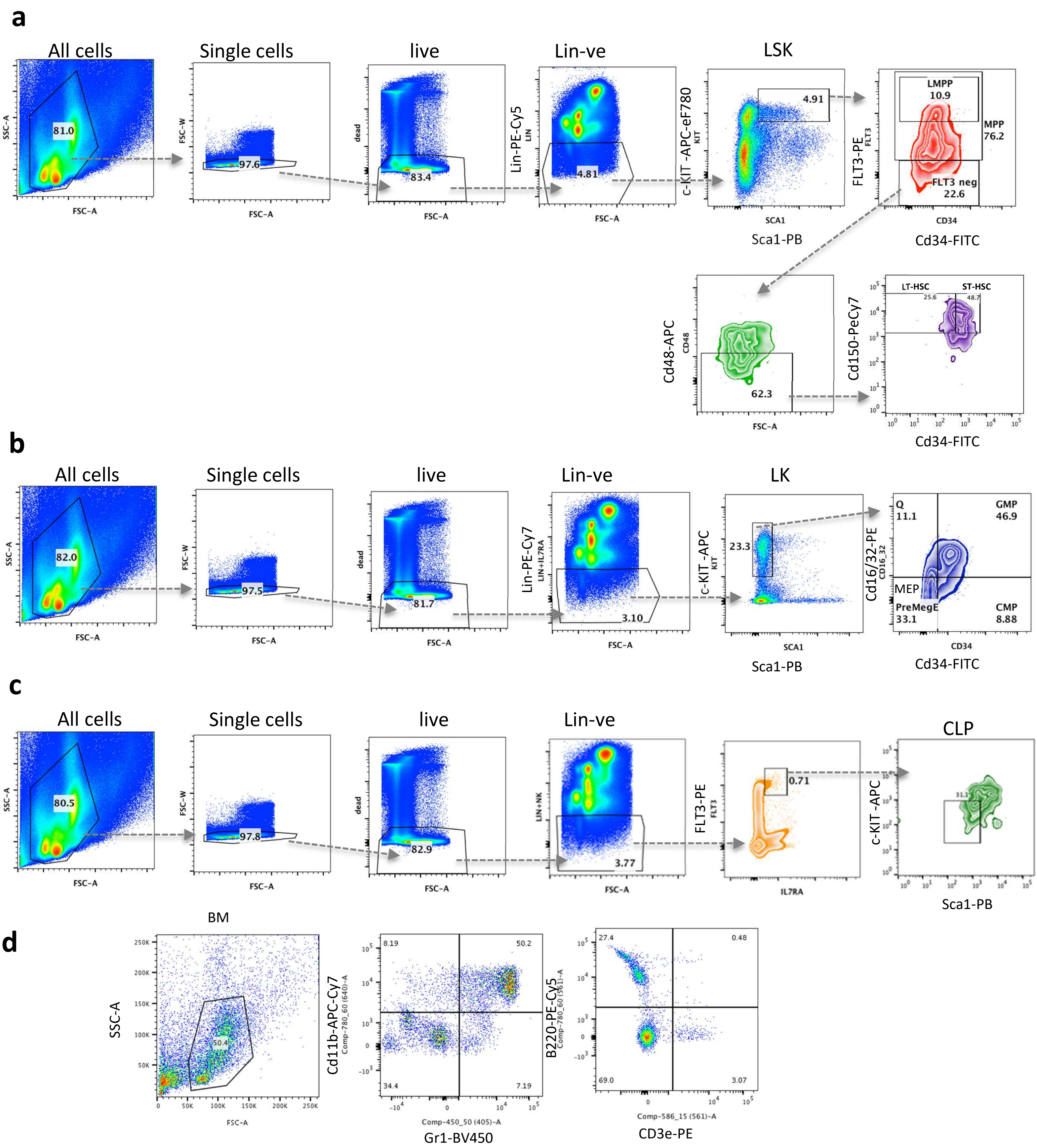

Supplementary Figure 3. Flow cytometry gating strategies for progenitor and differentiated blood panels shown in Extended Data Figure 1.

(**a**) Gating strategy for progenitor compartment shown in Extended Data Fig.1c-g, l-m (**b**) Gating strategy for LK, GMP, MEP and CMP shown in Extended Data Fig.1k. (**c**) CLP gating strategy in Extended Data Fig.1 g, n. (**d**) Gating strategy for differentiated cells used in Extended Data Figure 1h-j. Myeloid cells were defined as Mac1 and/or Gr1 positive; T cells as CD3 positive, B cells as B220 positive.

Supplementary Figure 4

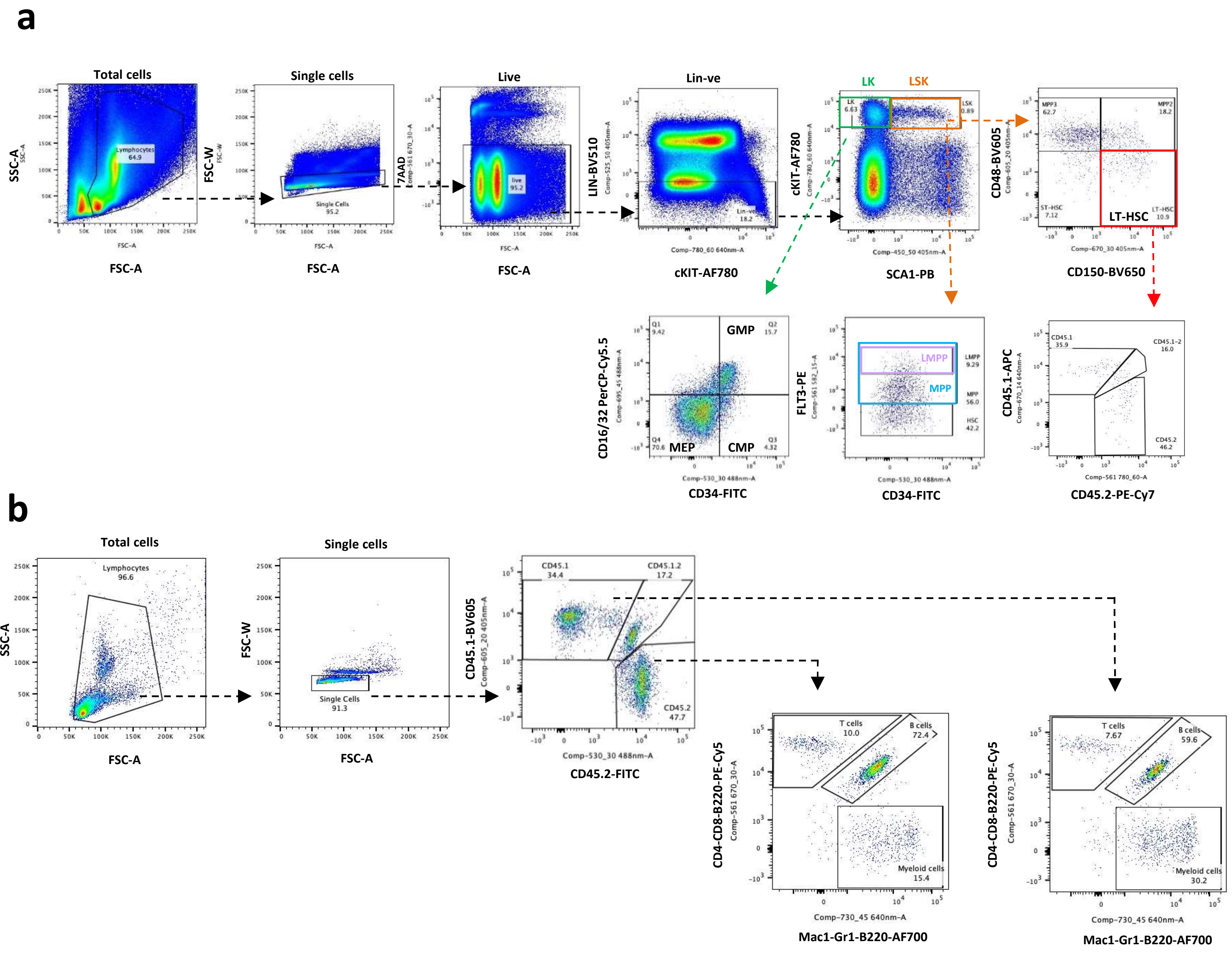

Supplementary Figure 4. Flow cytometry gating strategies for progenitor and differentiated blood panels shown in Figure 1, 4 and Extended Data Figure 2,6-7.

(**a**) LT-HSC and progenitor flow cytometry gating strategy used for transplanted mice in Fig.1g, Fig.4 b-j and Extended Data Figures: 2c, 6b-g, 6p-s, 7i-j, 7l-o (**b**) Flow cytometry gating strategy for B, T and Myeloid cells in PB, SP, BM used in transplant experiment. PB data are shown. The gating strategy was used in Extended Data Figures: 6h-k, 7a-d, 7k, 7q.

Supplementary Figure 5

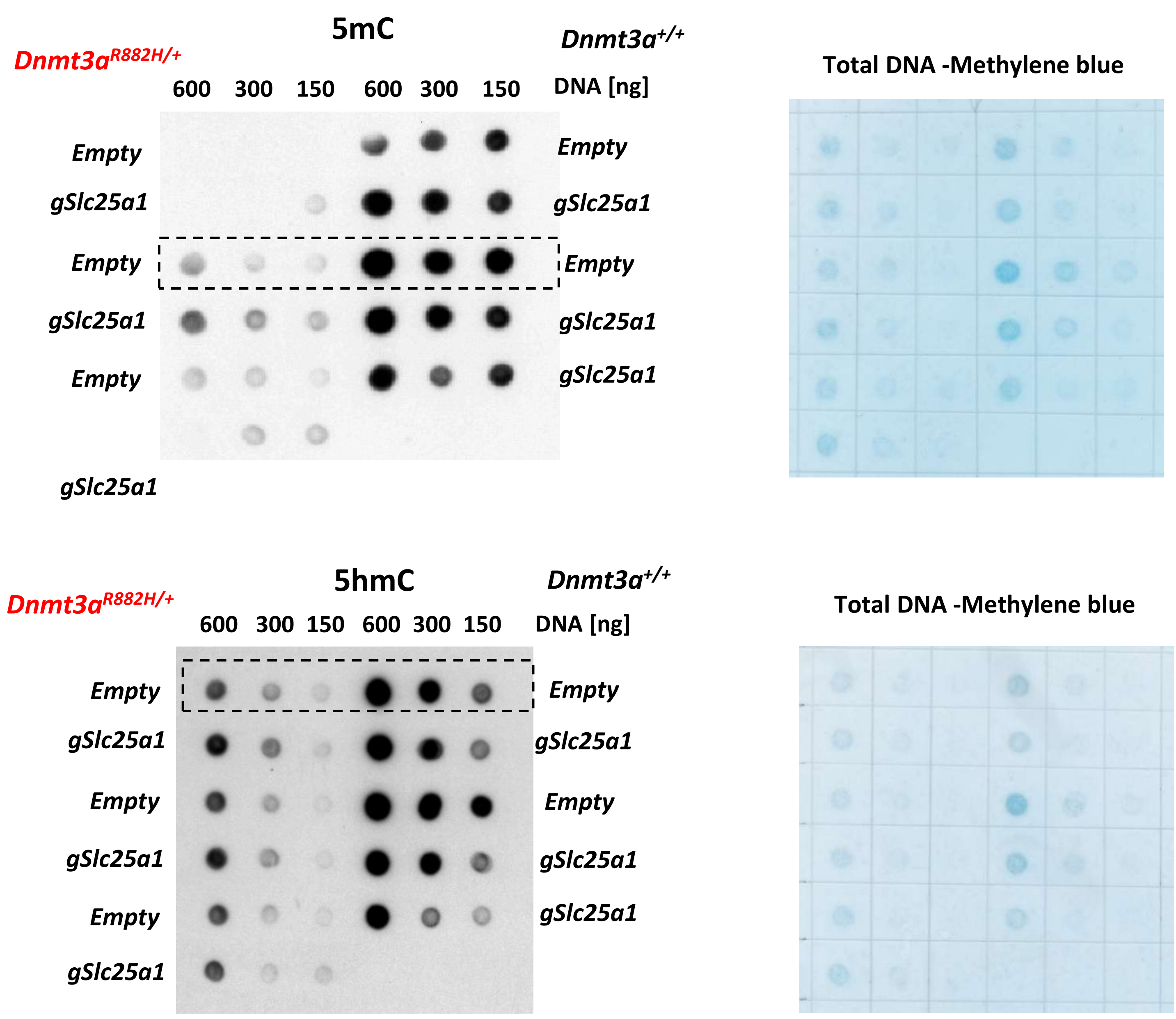

Supplementary Figure 5. Unprocessed dot blot images for Extended data Fig.8e

Unprocessed dot blot images are shown on the left, with corresponding total DNA image (DNA loading control – methylene blue staining) for each blot on the right. Cropping strategy is indicated with a dashed line.

Supplementary Figure 6

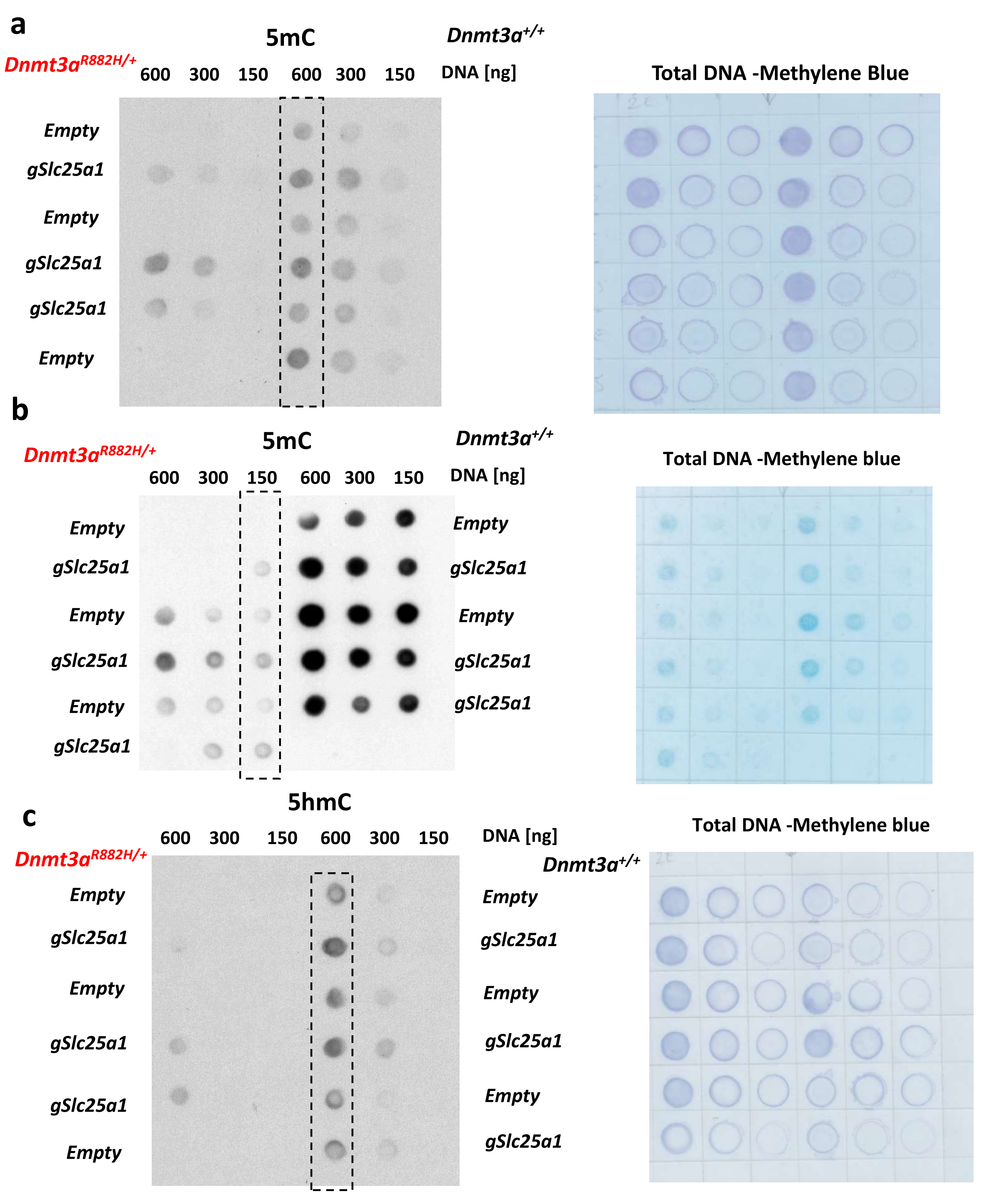

Supplementary Figure 6. Unprocessed dot blot images for Extended data Fig.8h, i, j
(**a-c**) Unprocessed dot blot images are shown on the left, with corresponding total DNA image (DNA loading control – methylene blue staining) for each blot on the right. Cropping strategy is indicated with a dashed line. Data correspond to (**a**) Extended data Fig.8h (**b**) Extended data Fig.8i (**c**) Extended data Fig.8j.

Supplementary Figure 7

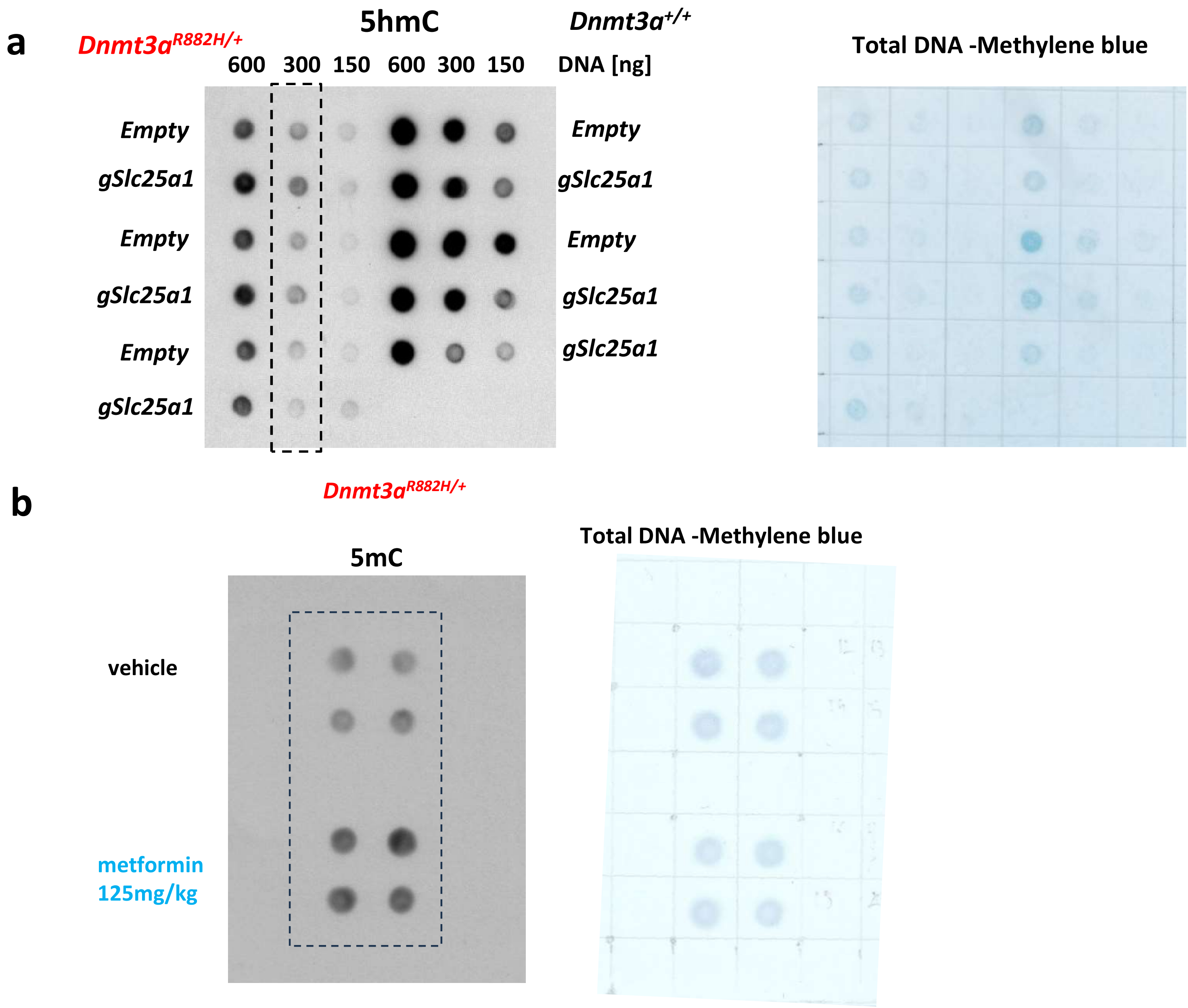

Supplementary Figure 7. Unprocessed dot blot images for Extended data Fig.8k and 9b
(**a-b**) Unprocessed dot blot images are shown on the left, with corresponding total DNA image (DNA loading control – methylene blue staining) for each blot on the right. Cropping strategy is indicated with a dashed line. Data correspond to (**a**) Extended data Fig.8k (**b**) Extended data Fig.9b.

Supplementary Figure 8

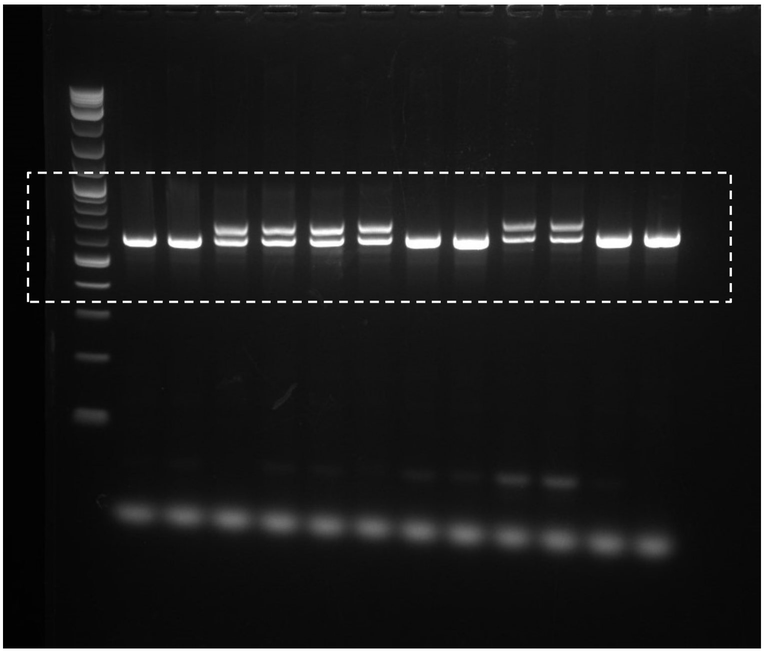

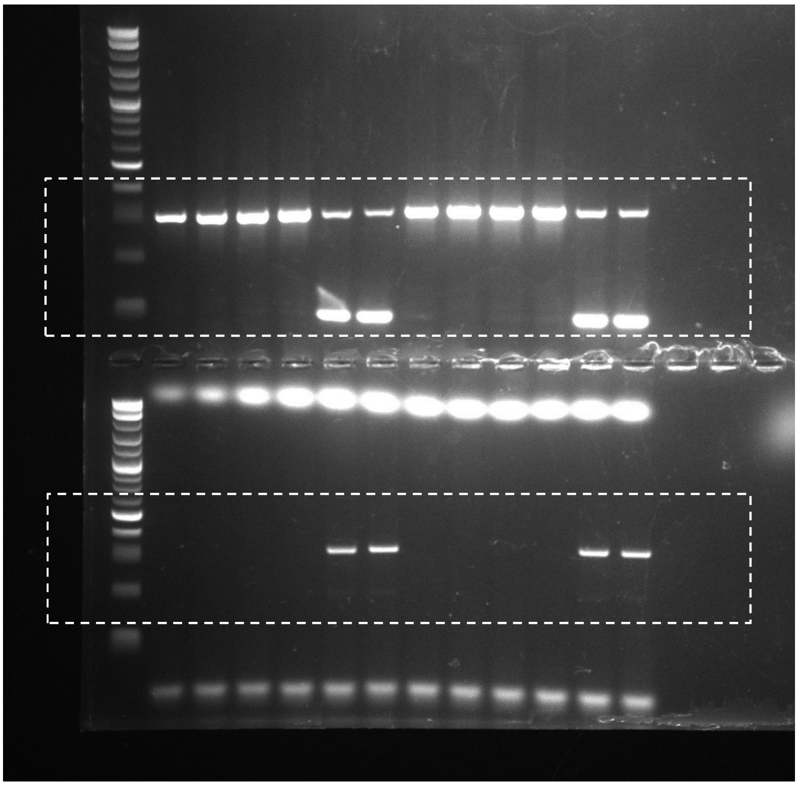

Supplementary Figure 8. Unprocessed DNA gel images for Extended data Fig.1b
Unprocessed DNA gel images corresponding to Extended data Fig.1b. Cropping strategy is indicated with dashed line.
