# Supplementary material for: Mitochondrial metabolism sustains DNMT3A-R882-mutant clonal haematopoiesis
Source: Nature. 2025 Apr 16;642(8067):431–41. doi: 10.1038/s41586-025-08980-6 (PMC12158785; doi:10.1038/s41586-025-08980-6)
Supplement: Supplementary file 2 — Reporting Summary [file 41586_2025_8980_MOESM2_ESM.pdf]

nature portfolio

Corresponding author(s): Malgorzata Gozdecka
Brian Huntly
George Vassiliou

Last updated by author(s): 02/03/2025

Reporting Summary

Nature Portfolio wishes to improve the reproducibility of the work that we publish. This form provides structure for consistency and transparency in reporting. For further information on Nature Portfolio policies, see our Editorial Policies and the Editorial Policy Checklist.

Statistics

For all statistical analyses, confirm that the following items are present in the figure legend, table legend, main text, or Methods section.

n/a	Confirmed	
☐	☒	The exact sample size (*n*) for each experimental group/condition, given as a discrete number and unit of measurement
☐	☒	A statement on whether measurements were taken from distinct samples or whether the same sample was measured repeatedly
☐	☒	The statistical test(s) used AND whether they are one- or two-sided<br>*Only common tests should be described solely by name; describe more complex techniques in the Methods section.*
☐	☒	A description of all covariates tested
☐	☒	A description of any assumptions or corrections, such as tests of normality and adjustment for multiple comparisons
☐	☒	A full description of the statistical parameters including central tendency (e.g. means) or other basic estimates (e.g. regression coefficient) AND variation (e.g. standard deviation) or associated estimates of uncertainty (e.g. confidence intervals)
☒	☐	For null hypothesis testing, the test statistic (e.g. *F*, *t*, *r*) with confidence intervals, effect sizes, degrees of freedom and *P* value noted<br>*Give P values as exact values whenever suitable.*
☒	☐	For Bayesian analysis, information on the choice of priors and Markov chain Monte Carlo settings
☒	☐	For hierarchical and complex designs, identification of the appropriate level for tests and full reporting of outcomes
☒	☐	Estimates of effect sizes (e.g. Cohen's *d*, Pearson's *r*), indicating how they were calculated

Our web collection on statistics for biologists contains articles on many of the points above.

Software and code

Policy information about availability of computer code

Data collection

- CRISPR sequencing was performed with HiSeq2500 system, Illumina.
- BD FACSDiva Software v9.0
- Histology slide scanner: Leica Aperio Slide Scanner AT20, running AperioServiceManager version 12.4.3.5008.
- LC-MS was performed with a Thermo Fisher Scientific Vanquish Horizon UHPLC System coupled to a Q Exactive Plus MS (Orbitrap MS 2.11 build 3006). The following software applications were used: Foundation 3.1 SP6, FreeStyle 1.5, Xcalibur 4.2 SP1 (version 4.2.47) and Thermo Scientific SII for Xcalibur (version 1.5.0.10747)
- Whole-exome sequencing reads of AML and control samples were mapped to the mouse genome assembly GRCm39 using BWA version 0.7.18 (arXiv:1303.3997 [q-bio.GN]) under default parameters and duplicated reads were flagged using Samtools version 1.9

Data analysis

- FlowJo (version 10.8.2)
- Seahorse data analysis was performed with Wave (version 2.6.3)
- CRISPR screen data was analysed with MAGeCK statistical package (version 0.5.3)
- Survival analysis was performed with R package (version 3.5-8) https://CRAN.R-project.org/package=survival.
- R package (version 4.2.2): For modelling anti-diabetic medications and clonal hematopoiesis (CH) using logistic regression.
- LC-MS data analysis was performed with Thermo Scientific Xcalibur 4.1 (version 4.1.31.9)
- Data were analysed and graphed using GraphPad Prism version 10 (v10.4.1)
- IGV software was used for visualizing data shown in Fig.1b. (version 2.3.0)
- MR analyses were performed using TwoSampleMR (v0.5.6 R) package
- Somatic mutations were called by GATK Mutect2 version 4.587
- Mutation calls were annotated with Ensembl VEP version 112

- Dot blots were scanned and analysed using Image Studio Lite software version 5.2

For manuscripts utilizing custom algorithms or software that are central to the research but not yet described in published literature, software must be made available to editors and reviewers. We strongly encourage code deposition in a community repository (e.g. GitHub). See the Nature Portfolio guidelines for submitting code & software for further information.

Data

Policy information about availability of data

All manuscripts must include a data availability statement. This statement should provide the following information, where applicable:

- Accession codes, unique identifiers, or web links for publicly available datasets
- A description of any restrictions on data availability
- For clinical datasets or third party data, please ensure that the statement adheres to our policy

- CRISPR screen raw data have been deposited in Gene Expression Omnibus with accession number GSE259404.
- Whole-exome and targeted sequencing data are available in Sequence Read Archive BioProject PRJNA1160274.
-Metabolomic data were deposited into MetaboLight depository (https://www.ebi.ac.uk/metabolights/) with accession number: MTBLS12201
- TCGA-AML cohort (151 samples) were downloaded from the GDA data portal. GDA portal accession weblink: https://portal.gdc.cancer.gov/projects/TCGA-LAML
-For UKB, individual-level data are under controlled accessed to protect sensitive information of the study participants. Individual-level UKB data may be requested via application to the UKB. All whole-exome sequencing data described in our study are available to registered researchers through the UKB data access protocol. Exomes can be found in the UKB showcase portal: https://biobank.ndph.ox.ac.uk/showcase/label.cgi?id=170. Additional information about data access registration is available at https://www.ukbiobank.ac.uk/enable-your-research/register.
- Whole-exome sequencing reads of AML and control samples were mapped to the mouse genome assembly GRCm39 using BWA version 0.7.18 (arXiv:1303.3997 [q-bio.GN]) under default parameters and duplicated reads were flagged using Samtools version 1.9
- Enrichr online software webpage link: https://maayanlab.cloud/Enrichr/
- DGIdb webpage link: https://dgidb.org/
-DepMap, Broad (2019) webpage link: https://figshare.com/articles/dataset/DepMap_19Q4_Public/11384241/3
- Survival Analysis R package, version 3.5-8, weblink: https://CRAN.R-project.org/package=survival.
- Meta-Analyses of Glucose and Insulin-related traits Consortium (MAGIC) http://magicinvestigators.org/

Research involving human participants, their data, or biological material

Policy information about studies with human participants or human data. See also policy information about sex, gender (identity/presentation), and sexual orientation and race, ethnicity and racism.

Reporting on sex and gender	For UKB cohort, sex concordance was determined by comparing clinically-reported sex against chromosome X:Y consensus coding sequence coverage ratio. Only sex, but not gender, was reported by this study.<br>- Human DNMT3A-R882 CH who donated PB sample used in this study was 41 year old female
Reporting on race, ethnicity, or other socially relevant groupings	For the UK Biobank cohort, ancestry of study participants were determined using the peddy software. Europeans individuals from UKB were defined with peddy-inferred European probability of at least 95% and were used for regression analyses.<br>- The DNMT3A-R882 CH participant was of European ancestry.
Population characteristics	UKB is a prospective cohort of approximately 500,000 adults, aged between 40 to 70 years, with genetic (whole-genome sequencing, whole-exome sequencing, SNP array) and phenotypic, proteomic, and matobolomic data available. 54.4% women, 45.6% men. Details for UKB have been described in Szustakowski et al. (Nature Genetics, 2021) and Bycroft et al. (Nature, 2018).<br>- Human DNMT3A-R882 CH participant was 41 year old female
Recruitment	For the UKB cohort, participants were aged between 40 to 70 years, and recruited between 2006 and 2010. The UKB has a degree of healthy volunteer bias.<br>The DNMT3A-R882 CH carrier attends the Cambridge Clonal Haematopoiesis Clinic, where she was approached and recruited into this study in 2022.
Ethics oversight	-UKB study has approval from the North-West Multi-centre Research Ethics Committee (11/NW/0382).<br>-Ethical approval for human DNMT3A-R882 CH sample used this study was granted by the East of England (Cambridge East) Research Ethics Committee (REC reference: 24/EE/0116). Informed consent was provided by the participant.

Note that full information on the approval of the study protocol must also be provided in the manuscript.

Field-specific reporting

Please select the one below that is the best fit for your research. If you are not sure, read the appropriate sections before making your selection.

☒ Life sciences ☐ Behavioural & social sciences ☐ Ecological, evolutionary & environmental sciences

For a reference copy of the document with all sections, see nature.com/documents/nr-reporting-summary-flat.pdf

Life sciences study design

All studies must disclose on these points even when the disclosure is negative.

Sample size	Sample sizes were chosen based on power calculations of expected differences and prior experience with these types of experiments. Sample sizes of n=3 mice per genotype/cell culture were chosen for most experiments with the exception of flow cytometric analysis and mouse experiments where sample size was increased to account for variation between individuals or for the need to carry out experiments at more than one timepoint.
Data exclusions	No data were excluded from the analysis
Replication	Most experiments were replicated 2-3 times. Independent biological and technical replicates were used per experiment, exact information is included in each figure legend.
Randomization	Mice were allocated to the study groups by genotype. For transplantation experiments animals of the same sex and similar age range were randomly assigned to study groups. For in vitro experiments mouse samples were also selected by genotype.
Blinding	Although the investigators were not blinded to the genotype of the animals, the animal technicians who provided the animal care, supervision and identification of sick animals at humane endpoint (and therefore making the decision to sacrifice sick animals) were blinded in order to eliminate survival bias. For transplant experiments, flow cytometry data collection and analyses were performed blind (by assigning a number to each animal ID). For in vitro experiments analyses were performed in batches of animals from both test and control groups using numbers instead of genotypes at the time of data acquisition. For statistical analyses samples were grouped into test vs control.

Behavioural & social sciences study design

All studies must disclose on these points even when the disclosure is negative.

Study description	N/A
Research sample	*State the research sample (e.g. Harvard university undergraduates, villagers in rural India) and provide relevant demographic information (e.g. age, sex) and indicate whether the sample is representative. Provide a rationale for the study sample chosen. For studies involving existing datasets, please describe the dataset and source.*
Sampling strategy	*Describe the sampling procedure (e.g. random, snowball, stratified, convenience). Describe the statistical methods that were used to predetermine sample size OR if no sample-size calculation was performed, describe how sample sizes were chosen and provide a rationale for why these sample sizes are sufficient. For qualitative data, please indicate whether data saturation was considered, and what criteria were used to decide that no further sampling was needed.*
Data collection	*Provide details about the data collection procedure, including the instruments or devices used to record the data (e.g. pen and paper, computer, eye tracker, video or audio equipment) whether anyone was present besides the participant(s) and the researcher, and whether the researcher was blind to experimental condition and/or the study hypothesis during data collection.*
Timing	*Indicate the start and stop dates of data collection. If there is a gap between collection periods, state the dates for each sample cohort.*
Data exclusions	*If no data were excluded from the analyses, state so OR if data were excluded, provide the exact number of exclusions and the rationale behind them, indicating whether exclusion criteria were pre-established.*
Non-participation	*State how many participants dropped out/declined participation and the reason(s) given OR provide response rate OR state that no participants dropped out/declined participation.*
Randomization	*If participants were not allocated into experimental groups, state so OR describe how participants were allocated to groups, and if allocation was not random, describe how covariates were controlled.*

Ecological, evolutionary & environmental sciences study design

All studies must disclose on these points even when the disclosure is negative.

Study description	N/A
Research sample	*Describe the research sample (e.g. a group of tagged Passer domesticus, all Stenocereus thurberi within Organ Pipe Cactus National Monument), and provide a rationale for the sample choice. When relevant, describe the organism taxa, source, sex, age range and any manipulations. State what population the sample is meant to represent when applicable. For studies involving existing datasets, describe the data and its source.*
Sampling strategy	*Note the sampling procedure. Describe the statistical methods that were used to predetermine sample size OR if no sample-size calculation was performed, describe how sample sizes were chosen and provide a rationale for why these sample sizes are sufficient.*

Data collection	*Describe the data collection procedure, including who recorded the data and how.*
Timing and spatial scale	*Indicate the start and stop dates of data collection, noting the frequency and periodicity of sampling and providing a rationale for these choices. If there is a gap between collection periods, state the dates for each sample cohort. Specify the spatial scale from which the data are taken*
Data exclusions	*If no data were excluded from the analyses, state so OR if data were excluded, describe the exclusions and the rationale behind them, indicating whether exclusion criteria were pre-established.*
Reproducibility	*Describe the measures taken to verify the reproducibility of experimental findings. For each experiment, note whether any attempts to repeat the experiment failed OR state that all attempts to repeat the experiment were successful.*
Randomization	*Describe how samples/organisms/participants were allocated into groups. If allocation was not random, describe how covariates were controlled. If this is not relevant to your study, explain why.*
Blinding	*Describe the extent of blinding used during data acquisition and analysis. If blinding was not possible, describe why OR explain why blinding was not relevant to your study.*

Did the study involve field work? ☐ Yes ☒ No

Field work, collection and transport

Field conditions	N/A
Location	*State the location of the sampling or experiment, providing relevant parameters (e.g. latitude and longitude, elevation, water depth).*
Access & import/export	*Describe the efforts you have made to access habitats and to collect and import/export your samples in a responsible manner and in compliance with local, national and international laws, noting any permits that were obtained (give the name of the issuing authority, the date of issue, and any identifying information).*
Disturbance	*Describe any disturbance caused by the study and how it was minimized.*

Reporting for specific materials, systems and methods

We require information from authors about some types of materials, experimental systems and methods used in many studies. Here, indicate whether each material, system or method listed is relevant to your study. If you are not sure if a list item applies to your research, read the appropriate section before selecting a response.

Materials & experimental systems

n/a	Involved in the study	
☐	☒	Antibodies
☐	☒	Eukaryotic cell lines
☒	☐	Palaeontology and archaeology
☐	☒	Animals and other organisms
☒	☐	Clinical data
☒	☐	Dual use research of concern
☒	☐	Plants

Methods

n/a	Involved in the study	
☒	☐	ChIP-seq
☐	☒	Flow cytometry
☒	☐	MRI-based neuroimaging

Antibodies

Antibodies used

Antibody used for FACS:

B220-Biotin, BD Biosciences, #559971, clone: RA3-6B2, lot: 6169942
Ter119-Biotin, BD Biosciences, #559971, clone: TER-119, lot: 6169946
Mac1-Biotin, BD Biosciences, #559971, clone: M1/70, lot: 6169944
CD3-Biotin, BD Biosciences, #559971, clone: 145-2C11, lot: 6169941
Gr1-Biotin, BD Biosciences, #559971, clone: RB6-8C5, lot: 6169943
Sca1-PB, BioLegend, #122520, clone: E13-161.7, lot: B385332
c-kit AF780, Thermo Fisher Scientific, #47-1171-82, clone: 2138, lot: 2577317
CD48-BV605, BioLegend, #103441, clone: HM48-1, lot: B308985
CD150-BV650, BioLegend, #115932, clone: TC15-12F12.2, lot: B390003
Cd135 PE, BD Biosciences, #553842, clone: A2F10.1, lot: 1354615
Cd34 FITC, Thermo Fisher Scientific, #11-0431-82, clone: RAM34, lot: 2518336
Cd16/32 PerCP PeCy5.5, BioLegend, #101324, clone: 93, lot: B378771

CD45.1 APC, BioLegend, #110714, clone: A20, lot: B254042
CD45.2 Pe-Cy7, Thermo Fisher Scientific, #25-0454-82, clone: 104, lot: 2628857
CD45.1 BV605, BioLegend, #110738, clone: A20, lot: B386453
CD45.2 FITC, BioLegend, #109806, clone: 104, lot: B395596
CD4 Pe-Cy5, BioLegend, #100514, clone: RM4-5, lot: B341915
Cd8 Pe-Cy5, BioLegend, #100710, clone: 53-6.7, lot: B369730
B220 Pe-Cy5, BioLegend, #103210, clone: RA3-6B2, lot: B354953
Gr1 AF700 , BioLegend, #108422, clone: RB6-8C5, lot: B386943
Mac1 AF700 , BioLegend, #101222, clone: M1/70 , lot: B350819
B220AF700 , BioLegend, #103232, clone: RA3-6B2, lot: B375847
7 AAD , BioLegend, #420404, lot: B383019
streptavidin BV510, BioLegend, #405233, lot: B362883
CD4 (Biolegend, #100514, clone: RM4-5, lot: B171933)
CD5 (Biolegend, #100610, clone: 53-7.3, lot: B178256)
CD8a (Biolegend, # 100710, clone: 53-6.7, lot: B166422)
CD11b (Biolegend, #101210, clone: M1/70, lot: B167424)
B220 (Biolegend, #103210, clone: RA3-6B2, lot: E07569-1635)
TER-119 (Biolegend, #116210, clone: TER-119, lot: B169021)
GR-1 (Biolegend, #108410, clone: RB6-8C5, lot: B179158)
SCA-1 (Biolegend, #122520, clone: E13-161.7, lot: B174209)
CD117 (eBioscience, # 47-1171-82, clone: 2B8, lot: E08461-1637)
CD48 (Biolegend, #103411, clone: HM-48-1, lot: B214727)
CD150 (Biolegend, #115913, clone: TC15-12F12.2, lot: B172059)
CD34 (BD Pharmigen, #553733, clone: RAM34, lot: 4319145)
FLT3 (BD Pharmigen, #553842, clone: A2F10.1, lot: 6148682)
IL7Ra (Biolegend, #135008, clone: A7R34, lot: B190405)
IL7Ra (Biolegend, #121103, clone: SB/199, lot: B176923)
Streptavidin (Biolegend, #405206, lot: B182997)
CD16/32 (BD Pharmigen, #553145, clone: 2.4G2, lot: 3123812)
c-KIT (BioLegend, #105812, clone: 2B8, lot: B217855)
NK (LSBio, LS-C62548, clone: PK136, lot: 44694)
CD45 (BD Pharmigen, #563891, clone: 30-F11, lot: 4276708)
CD11b (BD Pharmigen, #557657, clone: M1/70, lot: 4275513)
GR1 (BD Pharmigen, #560603, clone: 1A8, lot: 4290881)
TER119 (BD Pharmigen, #557915, clone: TER-119, lot: 7100737)
CD3e (eBioscience, #12-0031-82, clone: 145-2C11, lot: 4308509)

Antibody used for dot blot:
anti-5hmC Rb antibody, Active Motif, #39769, Lot: 25922004-14
anti-5mC Ms antibody, Active Motif, #61479, Lot: 23264180-11
anti- rabbit HRP-conjugated secondary antibody, 111-035-003, Jackson ImmunoResearch Laboratories Inc., lot #162297
anti-mouse HRP-conjugated secondary antibody (115-035-146, Jackson ImmunoResearch Laboratories Inc., lot #154319

Validation

Antibodies used in this study were validated for this application by the manufactures. The antibodies were also previously validated and published in multiple studies. Additionally, for each experiment we had relevant controls to ensure correct interpretation of results.
Lineage biotin panel kit, BD Biosciences, #559971, containing:
B220-biotin, BD Biosciences, clone: RA3-6B2
Ter119-biotin, BD Biosciences, clone: TER-119
Mac1-biotin, BD Biosciences, clone: M1/70
CD3-biotin, BD Biosciences, clone: 145-2C11
Gr1-Biotin, BD Biosciences, clone: RB6-8C5

Validation, references:
Used in several publications (see https://www.citeab.com/kits/10160766-559971-bd-pharmingen-biotin-mouse-lineage-panel) amongst them:
Thambyrajah, R., Maqueda, M., et al. 2024 Nat Commun. 15:4673

Sca1-PB, BioLegend UK Ltd, #122520, clone: E13-161.7
Validation:
Used in several publications (see https://www.biolegend.com/en-gb/products/pacific-blue-anti-mouse-ly-6a-e-sca-1-antibody-3901) amongst them:
Herrejon Chavez F, et al. 2023. Nat Commun. 14:2290

c-kit AF780 (APC-Cy7), Thermo Fisher Scientific, #47-1171-82, clone: 2138
Validation:
Used in several publications (see https://www.thermofisher.com/antibody/product/CD117-c-Kit-Antibody-clone-ACK2-Monoclonal/47-1172-82?gclid=Cj0KCQiAwOe8BhCCARIsAGKeD54z_rzvl-2VH9ysxpN2DEUj4LLHMpejjip0d_eRqqhc0g-FPs2kCRwaAis9EALw_wcB&ef_id=Cj0KCQiAwOe8BhCCARIsAGKeD54z_rzvl-2VH9ysxpN2DEUj4LLHMpejjip0d_eRqqhc0g-FPs2kCRwaAis9EALw_wcB:G:s&s_kwcid=AL!3652!3!278870232429!!!g!!!1454324556!63404918784&cid=bid_pca_frg_r01_co_cp1359_pjt0000_bid00000_0se_gaw_dy_pur_con&gad_source=1) amongst them:
Kazuhito Naka, et al. 2020. Nat Commun. 11:4681.

CD48-BV605, BioLegend UK Ltd, #103441, clone: HM48-1
Validation:
Used in several publications (see https://www.biolegend.com/en-gb/products/apc-anti-mouse-cd48-antibody-3622)
amongst them:
Ahrends T, et al. 2021. Cell. 184:5715

CD150-BV650, BioLegend UK Ltd, #115932, clone: TC15-12F12.2
Validation:
Used in several publications (see https://www.biolegend.com/en-us/search-results/pe-anti-mouse-cd150-slam-antibody-1369?GroupID=BLG10572&gad_source=1&gclid=Cj0KCQiAwOe8BhCCARIsAGKeD57ooSjsYZvP0TzD7ECmnfOmEKwWC2HbAy84VC3B66AGYxBHWHGWq1YaArM3EALw_wcB)
amongst them:
Li CC, et al. 2022. Nat Commun. 13:346.

Cd135 PE, BD Biosciences, #553842, clone: A2F10.1
Validation:
Used in several publications (see https://www.bdbiosciences.com/en-gb/products/reagents/flow-cytometry-reagents/research-reagents/single-color-antibodies-ruo/pe-rat-anti-mouse-cd135.553842?tab=citations_references)
amongst them:
Xiao, M., Kondo, S., et al. 2023. Nat Commun. 14:8372

Cd34 FITC, Thermo Fisher Scientific, #11-0431-82, clone: RAM34
Validation:
Used in several publications (see https://www.biocompare.com/9776-Antibodies/11185354-CD34-Monoclonal-Antibody-RAM34-FITC-eBioscience-8482/?pda=9776%7C11185354_0_0%7C1529%7C6%7CCD34)
amongst them:
Harbour JC, et al. 2023. Cell reports, 28;42(2):112105.

Cd16/32 PerCP PeCy5.5, BioLegend UK Ltd, #101324, clone:93
Validation:
Used in several publications (see https://www.biolegend.com/en-gb/products/percp-cyanine5-5-anti-mouse-cd16-32-antibody-6165?GroupID=BLG9237)
amongst them:
McAlpine CS, et al. 2021. Nature. 595:701

CD45.1 APC, BioLegend UK Ltd, #110714, clone: A20
Validation:
Used in several publications (see https://www.biolegend.com/en-gb/products/apc-anti-mouse-cd45-1-antibody-2319?GroupID=BLG1933)
amongst them:
Oh J, et al. 2023. Nat Commun. 14:3278

CD45.2 Pe-Cy7, Thermo Fisher Scientific, #25-0454-82, clone: 104
Validation:
Used in several publications (see https://www.thermofisher.com/antibody/product/CD45-2-Antibody-clone-104-Monoclonal/25-0454-82)
amongst them:
Stefanie Scherer, et al. 2023. Nat Immunol. 24(3):501-515

CD45.1 BV605, BioLegend UK Ltd, #110738, clone: A20
Validation:
Used in several publications (see https://www.biolegend.com/en-gb/products/brilliant-violet-605-anti-mouse-cd45-1-antibody-7850)
amongst them:
Scherer S, et al. 2023. Nat Immunol. 24:501
CD45.2 FITC, BioLegend UK Ltd, #109806, clone: 104
Validation:
Used in several publications (see https://www.biolegend.com/en-gb/products/fitc-anti-mouse-cd45-2-antibody-6)
amongst them:
Rundberg Nilsson A, et al. 2023. iScience. 26:106341

CD4 Pe-Cy5, BioLegend UK Ltd, #100514, clone: RM4-5
Validation:
Used in several publications (see https://www.biolegend.com/en-gb/products/pe-cyanine5-anti-mouse-cd4-antibody-483)
amongst them:
Yu X, et al. 2020. Nat Commun. 11:1110

Cd8 Pe-Cy5, BioLegend UK Ltd, #100710, clone: 53-6.7
Validation:
Used in several publications (see https://www.biolegend.com/en-gb/products/pe-cyanine5-anti-mouse-cd8a-antibody-156)
amongst them:
Campisi L, et al. 2022. Nature. 606:945

B220 Pe-Cy5, BioLegend UK Ltd, #103210, clone: RA3-6B2
Validation:
Used in several publications (see https://www.biolegend.com/en-gb/products/pe-cyanine5-anti-mouse-human-cd45r-b220-

antibody-448?GroupID=GROUP658)
amongst them:
Rundberg Nilsson A, et al. 2023. iScience. 26:106341

Gr1 AF700, BioLegend UK Ltd, #108422, clone: RB6-8C5
Validation:
Used in several publications (see https://www.biolegend.com/en-gb/products/alexa-fluor-700-anti-mouse-ly-6g-ly-6c-gr-1-antibody-3390)
amongst them:
Fite BZ, et al. 2021. Sci Rep. 11:927.

Mac1 AF700, BioLegend UK Ltd, #101222, clone: M1/70
Validation:
Used in several publications (see https://www.biolegend.com/en-gb/products/alexa-fluor-700-anti-mouse-human-cd11b-antibody-3388)
amongst them:
Griffin GK, et al. 2023. Nature. 618:834

B220 AF700, BioLegend UK Ltd, #103232, clone: RA3-6B2
Validation:
Used in several publications (see https://www.biolegend.com/en-gb/products/alexa-fluor-700-anti-mouse-human-cd45r-b220-antibody-3408)
amongst them:
Hao J, et al. 2022. Cell Rep. 41:111804

7 AAD, BioLegend UK Ltd, #420404
Validation:
Used in several publications (see https://www.biolegend.com/en-gb/products/7-aad-viability-staining-solution-1649?GroupID=BLG13283)
amongst them:
George M, et al. 2023. Cells. 12: 1186

streptavidin BV510, BioLegend UK Ltd, #405233
Validation:
Used in several publications (see https://www.biolegend.com/en-gb/products/brilliant-violet-510-streptavidin-8140)
amongst them:
Yankova E, et al. 2021. Nature. 593:597

CD5, BioLegend UK Ltd, #100610, clone: 53-7.3
Validation:
Used in several publications (see https://www.biolegend.com/en-gb/products/pe-cyanine5-anti-mouse-cd5-antibody-161)
amongst them:
Campisi L, et al. 2022. Nature. 606:945

CD11b, BioLegend UK Ltd, #101210, clone: M1/70
Validation:
Used in several publications (see https://www.biolegend.com/en-gb/products/pe-cyanine5-anti-mouse-human-cd11b-antibody-350)
amongst them:
Zhong W, et al. 2023. Nat Commun. 14:491

TER-119, BioLegend UK Ltd, #116210, clone: TER-119
Validation:
Used in several publications (see https://www.biolegend.com/en-gb/products/pe-cyanine5-anti-mouse-ter-119-erythroid-cells-antibody-1868)
amongst them:
Rundberg Nilsson A, et al. 2023. iScience. 26:106341

GR-1, BioLegend UK Ltd, #108410, clone: RB6-8C5
Validation:
Used in several publications (see https://www.biolegend.com/en-gb/products/pe-cyanine5-anti-mouse-ly-6g-ly-6c-gr-1-antibody-461)
amongst them:
Nita A, et al. 2021. Cell Reports. 34(5):108688

CD48, BioLegend UK Ltd, #103411, clone: HM-48-1
Validation:
Used in several publications (see https://www.biolegend.com/en-gb/products/apc-anti-mouse-cd48-antibody-3622)
amongst them:
Gonçalves R, et al. 2023. iScience. 26:105972

CD150, BioLegend UK Ltd, #115913, clone: TC15-12F12.2
Validation:
Used in several publications (see https://www.biolegend.com/en-gb/products/pe-cyanine7-anti-mouse-cd150-slam-antibody-3056)
amongst them:
Muto T, et al. 2022. Cell Stem Cell. 29:298.

CD34, BD Pharmigen, #553733, clone: RAM34
Validation:
Used in several publications (see https://www.citeab.com/antibodies/2413422-553733-bd-pharmingen-fitc-rat-anti-mouse-cd34)
amongst them:
Cockburn, K., Annusver, K., et al. 2022. Nature cell Biology. (24)1692–1700

IL7Ra, BioLegend UK Ltd, #135008, clone: A7R34
Validation:
Used in several publications (see https://www.biolegend.com/en-gb/products/fitc-anti-mouse-cd127-il-7ralpha-antibody-6189?GroupID=BLG7953)
amongst them:
Dong J, et al. 2013. PLoS One. 8:e56378.

IL7Ra, BioLegend UK Ltd, #121103, clone: SB/199
Validation:
Used in several publications (see https://www.biolegend.com/en-gb/products/biotin-anti-mouse-cd127-il-7ralpha-antibody-3048?GroupID=BLG4697)
amongst them:
Gozdecka M, et al. 2018. Nat Genet. 50:883

Streptavidin, BioLegend UK Ltd, #405206
Validation:
Used in several publications (see https://www.biolegend.com/en-gb/products/pe-cyanine7-streptavidin-1477?GroupID=GROUP23)
amongst them:
Takano T, et al. 2023. Nat Commun. 14:1451

CD16/32, BD Pharmigen, #553145, clone: 2.4G2
Validation:
Used in several publications (see https://www.citeab.com/antibodies/2408169-553145-bd-pharmingen-pe-rat-anti-mouse-cd16-cd32)
amongst them:
Omatsu, Y., Aiba, S., et al. 2022. Nature Commun. 13: 2654

c-KIT, BioLegend UK Ltd, #105812, clone: 2B8
Validation:
Used in several publications (see https://www.biolegend.com/en-gb/products/apc-anti-mouse-cd117-c-kit-antibody-72)
amongst them:
Wang X, et al. 2023. Nature. 618:808

NK (LSBio, LS-C62548, clone: PK136, lot: 44694)
Validation:
Antibody used in several studies including:
Dovey O, et al., 2017, 2017 Oct 26;130(17):1911-1922

CD45, BD Pharmigen, #563891, clone: 30-F11
Validation:
Used in several publications (see https://www.citeab.com/antibodies/2407961-563891-bd-horizon-bv510-rat-anti-mouse-cd45)
amongst them:
Peruzzotti-Jametti, L., Willis, C. M., et al. 2024 Nature. 628: 195–203

CD11b, BD Pharmigen, #557657, clone: M1/70
Validation:
Used in several publications (see https://www.citeab.com/antibodies/2406880-557657-bd-pharmingen-apc-cy-7-rat-anti-cd11b)
amongst them:
Natarajan, N., Florentin, J., et al. 2024. Nature Commun. 15:7337

GR1, BD Pharmigen, #560603, clone: 1A8
Validation:
Used in several publications (see https://www.citeab.com/antibodies/2408945-560603-bd-horizon-v450-rat-anti-mouse-ly-6g)
amongst them:
Mudalagiriyappa, S., Sharma, J., et al. 2022. Cell Reports. 41:111543

TER119, BD Pharmigen, #557915, clone: TER-119
Validation:
Used in several publications (see https://www.citeab.com/antibodies/2409130-557915-bd-pharmingen-fitc-rat-anti-mouse-ter-119-er)
amongst them:
Gerdes, P., Lim, S. M., et al. 2022. Nature Commun. 13: 7470

CD3e, eBioscience, #12-0031-82, clone: 145-2C11
Validation:
Used in several publications (see https://www.thermofisher.com/antibody/product/CD3e-Antibody-clone-145-2C11-Monoclonal/16-0031-82?gclid=CjwKCAiA2JG9BhAuEiwAH_zf3iDpTVfCXP3DlkOFJhHsH3kDPRZpkiku-q4as4OxtJymEJG47ld-jxoCecUQAvD_BwE&ef_id=CjwKCAiA2JG9BhAuEiwAH_zf3iDpTVfCXP3DlkOFJhHsH3kDPRZpkiku-q4as4OxtJymEJG47ld-jxoCecUQAvD_BwE:G:s&s_kwcid=AL!3652!3!278870232429!!!g!!!1454324556!63404918784&cid=bid_pca_frg_r01_co_cp1359_pjt0000_bid00000_0se_gaw_dy_pur_con&gad_source=1)
amongst them:
Vera-Marie E Dunlock, et al. 2022. Cell Reports. 13:111006

anti-5mC Ms antibody, Active Motif, #61479
Validation:
The antibody has been validated for use in dot blot application (please see https://www.activemotif.com/catalog/details/61479/5-methylcytosine-5-mc-antibody-mab-clone-a1).

anti-5hmC Rb antibody, Active Motif, #39769
Validation:
The antibody has been validated for use in dot blot application (see https://www.activemotif.com/catalog/details/39769 and used in several studies including:
Huang et al. 2019. Cancer Cell Apr 15;35(4):677-691.e10.

anti- rabbit HRP-conjugated secondary antibody, 111-035-003, Jackson ImmunoResearch Laboratories Inc.,
Validation:
Antibody used in sever publications, please see: https://www.biocompare.com/9776-Antibodies/5606382-Peroxidase-AffiniPure-Goat-Anti-Rabbit-IgG-H-L/

anti-mouse HRP-conjugated secondary antibody (115-035-146, Jackson ImmunoResearch Laboratories Inc.,
Validation:
Antibody used in sever publications, please see: https://www.jacksonimmuno.com/catalog/products/115-035-146/Goat-Mouse-IgG-HL-Horseradish-Peroxidase.

Eukaryotic cell lines

Policy information about cell lines and Sex and Gender in Research

Cell line source(s)	293-FT (Invitrogen, R70007)
Authentication	Purchase from the provider (Invitrogen) as an authenticated cell line. We did not performed any further authentication and used early passages (p<10) for lentiviral vector production.
Mycoplasma contamination	Cell line was tested negative for Mycoplasma
Commonly misidentified lines (See ICLAC register)	No commonly misidentified cells were used

Palaeontology and Archaeology

Specimen provenance	N/A
Specimen deposition	*Indicate where the specimens have been deposited to permit free access by other researchers.*
Dating methods	*If new dates are provided, describe how they were obtained (e.g. collection, storage, sample pretreatment and measurement), where they were obtained (i.e. lab name), the calibration program and the protocol for quality assurance OR state that no new dates are provided.*

☐ Tick this box to confirm that the raw and calibrated dates are available in the paper or in Supplementary Information.

Ethics oversight	*Identify the organization(s) that approved or provided guidance on the study protocol, OR state that no ethical approval or guidance was required and explain why not.*

Note that full information on the approval of the study protocol must also be provided in the manuscript.

Animals and other research organisms

Policy information about studies involving animals; ARRIVE guidelines recommended for reporting animal research, and Sex and Gender in Research

Laboratory animals	New Dnmt3aR882H/+ mouse model was constructed by flanking native mouse exon 23 of Dnmt3a with loxP sites and introducing human exon 23 containing DNMT3A-R882 mutation. PGK-Puro cassette flanked with Rox sites was inserted after the human exon 23. Mx1-Cre mouse model was reported previously63. Cas9-expressing mice were reported previously22. Mice were analyzed at different age time points, which are specified in the corresponding figure legends/result section. Dnmt3aR882H/+ between 6-12 week of age as well as one year post pIpC were used for characterization of bone marrow compartment by FACS, this information is provided in the figure legend. For transplantation experiment Dnmt3aR882H/+, aged matched competitor and WT were used at age between 6-12 post pIpC. For isolation of cells for screens and for screen validation Dnmt3aR882H/+, Cas9 and WT,Cas9 mice were collected 6-8 weeks post pIpC.<br>B6.SJL-Ptprca Pepcb/BoyJ mice (CD45.1, Jackson Laboratory #002014). Wild type mice: C57BL/6J (CD45.2, Jackson Laboratory #000664). CD45.1/CD45.2 recipient mice, generated by crossing CD45.1 and CD45.2 mice, and were used in this study were between 8 to 18 weeks of age. CD45.1 mice used as competitors for transplantation were age/sex matched to the test cells (e.g., CD45.2-Dnmt3aR882H/+). Mice were housed in specific pathogen-free conditions. All cages were on a 12:12-h light:dark cycle (lights on, 07:30) in a temperature-controlled and humidity-controlled room. Room temperature was maintained at 72 ± 2 °F (22.2 ± 1.1 °C), and room humidity was maintained at 30–70%.
Wild animals	No wild animals were used in this study.
Reporting on sex	Equal numbers of males and females were used in the study, except for transplant experiments which employed female recipients.
Field-collected samples	No field-collected samples were used in the study.
Ethics oversight	The in vivo experiments were performed under project license PPL 80/2564 and PP3797858 issued by the United Kingdom Home Office, in accordance with the Animal Scientific Procedures Act 1986. Murine ethical compliance was approved by the University of Cambridge Animal Welfare and Ethical Review Body

Note that full information on the approval of the study protocol must also be provided in the manuscript.

Clinical data

Policy information about clinical studies

All manuscripts should comply with the ICMJE guidelines for publication of clinical research and a completed CONSORT checklist must be included with all submissions.

Clinical trial registration	*Provide the trial registration number from ClinicalTrials.gov or an equivalent agency.*
Study protocol	*Note where the full trial protocol can be accessed OR if not available, explain why.*
Data collection	*Describe the settings and locales of data collection, noting the time periods of recruitment and data collection.*
Outcomes	*Describe how you pre-defined primary and secondary outcome measures and how you assessed these measures.*

Dual use research of concern

Policy information about dual use research of concern

Hazards

Could the accidental, deliberate or reckless misuse of agents or technologies generated in the work, or the application of information presented in the manuscript, pose a threat to:

No	Yes	
☒	☐	Public health
☒	☐	National security
☒	☐	Crops and/or livestock
☒	☐	Ecosystems
☒	☐	Any other significant area

Experiments of concern

Does the work involve any of these experiments of concern:

No	Yes	
☒	☐	Demonstrate how to render a vaccine ineffective
☒	☐	Confer resistance to therapeutically useful antibiotics or antiviral agents
☒	☐	Enhance the virulence of a pathogen or render a nonpathogen virulent
☒	☐	Increase transmissibility of a pathogen
☒	☐	Alter the host range of a pathogen
☒	☐	Enable evasion of diagnostic/detection modalities
☒	☐	Enable the weaponization of a biological agent or toxin
☒	☐	Any other potentially harmful combination of experiments and agents

Plants

Seed stocks: N/A

Novel plant genotypes: *Describe the methods by which all novel plant genotypes were produced. This includes those generated by transgenic approaches, gene editing, chemical/radiation-based mutagenesis and hybridization. For transgenic lines, describe the transformation method, the number of independent lines analyzed and the generation upon which experiments were performed. For gene-edited lines, describe the editor used, the endogenous sequence targeted for editing, the targeting guide RNA sequence (if applicable) and how the editor was applied.*

Authentication: *Describe any authentication procedures for each seed stock used or novel genotype generated. Describe any experiments used to assess the effect of a mutation and, where applicable, how potential secondary effects (e.g. second site T-DNA insertions, mosiacism, off-target gene editing) were examined.*

ChIP-seq

Data deposition

☐ Confirm that both raw and final processed data have been deposited in a public database such as GEO.

☐ Confirm that you have deposited or provided access to graph files (e.g. BED files) for the called peaks.

Data access links
May remain private before publication.: *For "Initial submission" or "Revised version" documents, provide reviewer access links. For your "Final submission" document, provide a link to the deposited data.*

Files in database submission: *Provide a list of all files available in the database submission.*

Genome browser session (e.g. UCSC): *Provide a link to an anonymized genome browser session for "Initial submission" and "Revised version" documents only, to enable peer review. Write "no longer applicable" for "Final submission" documents.*

Methodology

Replicates: *Describe the experimental replicates, specifying number, type and replicate agreement.*

Sequencing depth: *Describe the sequencing depth for each experiment, providing the total number of reads, uniquely mapped reads, length of reads and whether they were paired- or single-end.*

Antibodies: *Describe the antibodies used for the ChIP-seq experiments; as applicable, provide supplier name, catalog number, clone name, and lot number.*

Peak calling parameters: *Specify the command line program and parameters used for read mapping and peak calling, including the ChIP, control and index files used.*

Data quality: *Describe the methods used to ensure data quality in full detail, including how many peaks are at FDR 5% and above 5-fold enrichment.*

Software: *Describe the software used to collect and analyze the ChIP-seq data. For custom code that has been deposited into a community repository, provide accession details.*

Flow Cytometry

Plots

Confirm that:

☒ The axis labels state the marker and fluorochrome used (e.g. CD4-FITC).

☒ The axis scales are clearly visible. Include numbers along axes only for bottom left plot of group (a 'group' is an analysis of identical markers).

☒ All plots are contour plots with outliers or pseudocolor plots.

☒ A numerical value for number of cells or percentage (with statistics) is provided.

Methodology

Sample preparation	This information is provided in the supplementary method section.
Instrument	Flow cytometry analysis was performed using the LSRFortessa instrument (BD).
Software	Flow cytometry analysis was performed using a LSRFortessa instrument and resulting data were subsequently analysed using FlowJo (version 10.8.2).
Cell population abundance	The purity of the relevant cells was over 98% in the post-sort fraction as assessed by flow cytometry.
Gating strategy	Gating strategy is provided in Extended Data Fig. 6e-f

☒ Tick this box to confirm that a figure exemplifying the gating strategy is provided in the Supplementary Information.

Magnetic resonance imaging

Experimental design

Design type	*Indicate task or resting state; event-related or block design.*
Design specifications	*Specify the number of blocks, trials or experimental units per session and/or subject, and specify the length of each trial or block (if trials are blocked) and interval between trials.*
Behavioral performance measures	*State number and/or type of variables recorded (e.g. correct button press, response time) and what statistics were used to establish that the subjects were performing the task as expected (e.g. mean, range, and/or standard deviation across subjects).*

Acquisition

Imaging type(s)	*Specify: functional, structural, diffusion, perfusion.*
Field strength	*Specify in Tesla*
Sequence & imaging parameters	*Specify the pulse sequence type (gradient echo, spin echo, etc.), imaging type (EPI, spiral, etc.), field of view, matrix size, slice thickness, orientation and TE/TR/flip angle.*
Area of acquisition	*State whether a whole brain scan was used OR define the area of acquisition, describing how the region was determined.*

Diffusion MRI ☐ Used ☒ Not used

Preprocessing

Preprocessing software	*Provide detail on software version and revision number and on specific parameters (model/functions, brain extraction, segmentation, smoothing kernel size, etc.).*
Normalization	*If data were normalized/standardized, describe the approach(es): specify linear or non-linear and define image types used for transformation OR indicate that data were not normalized and explain rationale for lack of normalization.*
Normalization template	*Describe the template used for normalization/transformation, specifying subject space or group standardized space (e.g. original Talairach, MNI305, ICBM152) OR indicate that the data were not normalized.*
Noise and artifact removal	*Describe your procedure(s) for artifact and structured noise removal, specifying motion parameters, tissue signals and physiological signals (heart rate, respiration).*
Volume censoring	*Define your software and/or method and criteria for volume censoring, and state the extent of such censoring.*

Statistical modeling & inference

Model type and settings

Specify type (mass univariate, multivariate, RSA, predictive, etc.) and describe essential details of the model at the first and second levels (e.g. fixed, random or mixed effects; drift or auto-correlation).

Effect(s) tested

Define precise effect in terms of the task or stimulus conditions instead of psychological concepts and indicate whether ANOVA or factorial designs were used.

Specify type of analysis: ☐ Whole brain ☐ ROI-based ☐ Both

Statistic type for inference

(See Eklund et al. 2016)

Specify voxel-wise or cluster-wise and report all relevant parameters for cluster-wise methods.

Correction

Describe the type of correction and how it is obtained for multiple comparisons (e.g. FWE, FDR, permutation or Monte Carlo).

Models & analysis

n/a	Involved in the study
☐	☐ Functional and/or effective connectivity
☐	☐ Graph analysis
☐	☐ Multivariate modeling or predictive analysis

Functional and/or effective connectivity

Report the measures of dependence used and the model details (e.g. Pearson correlation, partial correlation, mutual information).

Graph analysis

Report the dependent variable and connectivity measure, specifying weighted graph or binarized graph, subject- or group-level, and the global and/or node summaries used (e.g. clustering coefficient, efficiency, etc.).

Multivariate modeling and predictive analysis

Specify independent variables, features extraction and dimension reduction, model, training and evaluation metrics.
