# Supplementary material for: Mitochondrial metabolism sustains DNMT3A-R882-mutant clonal haematopoiesis
Source: Nature. 2025 Apr 16;642(8067):431–41. doi: 10.1038/s41586-025-08980-6 (PMC12158785; doi:10.1038/s41586-025-08980-6)
Supplement: Supplementary file 4 — Supplementary Tables 1–11. [file 41586_2025_8980_MOESM4_ESM.zip › 2024-02-04132E-s4/SupplementaryTableLegends_ESM.docx]

**Supplementary information guide**

**Supplementary Tables**

**Supplementary Table 1.** **Exome sequencing data of MPN/AML *Dnmt3a^R882H/+^* compared to normal control.** This file contains exome sequencing data analysis of six mouse *Dnmt3a^R882H/+^* MPN/AMLs compared to normal, untransformed sample.

**Supplementary Table 2.** **Identified *Dnmt3a^R882H/+^* vulnerabilities.** This file contains the list of all vulnerability genes for *Dnmt3a^R882H/+^* HSPCs identified on day 30 of the CRISPR screen at FDR <20%.

**Supplementary Table 3. *Dnmt3a^R882H/+^* vulnerabilities not present in HPC-7.** This file contains a list of *Dnmt3a^R882H/+^*-specific vulnerabilities. This list was generated by removing vulnerabilities present in HPC-7 cell line from the *Dnmt3a^R882H/+^* list.

**Supplementary Table 4.** **List of druggable categories of *Dnmt3a^R882H/+^* specific dropouts by DGIdb.** This file contains functional classification of *Dnmt3a^R882H/+^*-specific vulnerabilities performed with DGIdb.

**Supplementary Table 5.** **Drug-gene interaction categories for *Dnmt3a^R882H/+^* specific dropouts identified by DGIdb.** This file provides a drug-gene interaction output from DGIdb for *Dnmt3a^R882H/+^*-specific vulnerability genes.

**Supplementary Table 6.** **Logistic regression summary statistics for the association between CH (outcome) and anti-diabetic medication (predictor).** This file contains logistic regression summary statistics for the association between CH (outcome) and anti-diabetic medication (predictor) adjusted for age, sex, smoking status, and the first four genetic principal components. Related to Fig. 5b and c and Extended Data Fig. 11a, c and d.

**Supplementary Table 7.** **Logistic regression summary statistics for the association between CH (outcome) and surrogate markers for undiagnosed or untreated diabetes at recruitment (predictor).** This file contains logistic regression summary statistics for the association between CH (outcome) and surrogate markers for undiagnosed or untreated diabetes at recruitment (predictor), adjusted for age, sex, smoking status, and the first four genetic principal components. Related to Fig. 5d and e.

**Supplementary Table 8.** **Inverse variance weighting (IVW) summary statistics from Mendelian randomisation of genetic instruments of glycaemic-related traits (exposure) on CH (outcome).** IVW summary statistics from Mendelian randomisation of genetic instruments of glycaemic-related traits (exposure) on CH (outcome). Related to Extended Data Fig. 11c-f.

**Supplementary Table 9.** **Genotyping primers used in the study.** Sequence of primers used for genotyping of *Mx1-Cre*, *Dnmt3a^R882H/+^* and recombined *Dnmt3a^R882H/+^* alleles. Expected product sizes are also provided.

**Supplementary Table 10.** **Sequence of primers used for gRNA cloning used for validation studies.**

This file contains sequences of primers used for cloning of selected gRNAs.

**Supplementary Table 11.** **LC-MS measurement -LC gradient information.** These data contain LC gradient used for (**a**) the C18pfp column and (**b**) the HSST3 column used for LC-MS measurements described in the Methods section.
